# Using multivariate statistical methods to assess the urban smartness on the example of selected European cities

**DOI:** 10.1371/journal.pone.0240260

**Published:** 2020-12-23

**Authors:** Sławomira Hajduk

**Affiliations:** Faculty of Engineering Management, Bialystok University of Technology, Białystok, Poland; Institute for Advanced Sustainability Studies, GERMANY

## Abstract

The growing importance of maturity smart cities is currently observed worldwide. The vast majority of smart city models focus on hard domains such as communication and technology infrastructure. Scientists emphasize the need to take into account social capital and the knowledge of residents. The smart cities invest in enhanced openness and transparency data. Mature smart cities use real-time evidences and information to citizens, businesses and visitors. The smart cities are characterized by bottom-down management and civil government. The paper aims to assess the urban smartness of selected European cities based on the ISO 37120 standard. Several research methods including the Multidimensional Statistical Analysis (MSA) were applied. Using the statistical analysis of European smart cities with the implemented ISO 37120 standard, the author tried to fill gaps in the knowledge and to evaluate maturity smart cities. The results of the research have shown that the smart city concept is a viable strategy which contributes to the urban sustainability. The author also found out that urban sustainability frameworks contain a large number of indicators measuring environmental sustainability, the smart city frameworks lack environmental indicators while highlighting social and economic aspects.

## Introduction

Cities play an extensive role in the sustainable development of the world. Cities constitute centers of innovation, entrepreneurship and creativity. According to United Nations, 56% of the world’s population resides in cities, whereas forecasts indicate an increase to 69% in 2050. In fact, the world’s 750 largest cities generate 57% of global GDP [1]. On the other hand, cities face many challenges, including overpopulation, environmental pollution and social segregation. Cities emit over 70% of global greenhouse gases and consume 80% of the world’s energy. The European Union assumes that cities will reduce greenhouse gas emissions by 60% to 2050 [2]. Contemporary urban development is increasingly focused on ICT and sustainability in the so-called smartization process. Author’s smart city definition refers to Fernandez-Anez’s definition [3]. Smart city means a system achieves sustainable development and a high quality of life using ICT infrastructure.

The main purpose of the article is to attempt the assessment of urban smartness for selected European cities. The manuscript aims to show dependences occurring between the theoretical and practical considerations concerning cities based on the difficult implementation of the urban smartness. Firstly, the paper serves to organize the terminology in the field of sustainability and urban smartness in the smart cities concept and proposes smart city definition. The literature available on the Web of Science, Springer, Scopus, IEEE and Elsevier databases has been reviewed. Secondly, the article is concerned with reflection on sustainable city often confronted with the smart city concept. The empirical part attempts to assess the urban smartness indicators for selected European cities with the implemented ISO 37120 standard through the use of the cluster analysis and the factor analysis. The manuscript is an attempt to answer the research questions: how can we measure the urban smartness and which variables, which cities have the highest level of urban smartness, how does classification of cities present in terms of urban smartness and how are the dependencies between pillars of sustainability?

Smart city is a new method of urban management. According to this concept ICT is used to improve the life quality of residents and urban services. Since there is no research on development of urban management, the article focuses on identifying European cities most involved in implementing the concept of smart city. The article attempts to fill this research gap.

In world literature there are many definitions of a smart city. Hollands [4] suggests that a smart city helps to solve problems related to urbanization, in particular environmental pollution, land consumption, urban sprawl, traffic congestion, energy needs. On the other hand, Harrison’s *et al*. [5] approach describes a smart city as an instrumented and interconnected. According to Nam and Pardo [6], the concept of a smart city is linked to such fundamental components as: (I) technology factors: physical infrastructure, smart technologies, mobile technologies, virtual technologies, digital technologies; (II) human factors: social infrastructure, learning capital; (III) institutional factors: governance, policy, regulations. In turn, Lombardi’s *et al*. [7] identified entrepreneurship and innovation as the most important features of contemporary urban development. Giffinger *et al*. [8] distinguished six dimensions such as: economy, mobility, environment, living, governance, people.

Furthermore, Cocchia [9] prepared a bibliometric analysis of publications on smart city. Smart cities invest in human and social capital as well as use ICT to conduct sustainability and improve the life quality [10]. Cities managed in accordance with the guidelines of smart cities concept effectively solve social and environmental problems [11]. Cities determine the perspectives of all components of the urban settlement unit by building intelligent connections between self- decisive, independent and conscious citizens [3]. The smart city concept obtains three paradigms: (I) a digital city, using ICT to support and create cooperation networks for citizens and organizations, sharing data and information, and combining online service such as e-administration and e-democracy [12]; (II) a knowledge city, based on the enforcement and valuation of data and information available and produced in the city [13]; (III) a green city, concerns an ecological vision of urban space based on sustainability and reduction of the city’s footprint in the environment [14]. Worldwide, there are a lot of criterions classification smart city such as goals, dimensions, generations, paradigms (Table 1).

**Table 1:** Classification of terms in academic Smart City definitions.

Criterions	Goals	Dimensions	Generations	Paradigms
		Environment		
	Sustainability	Economy	Smart City 1.0	Digital city
**Classes**	Quality of life	Mobility	Smart City 2.0	Green city
	Efficiency	People	Smart City 3.0	Knowledge city
		Living		
		Governance		

Note: author’s elaboration on the based [11, 15–21].

Wiseman believes that urban management is a complex task because of urbanization and climate change [22]. Cities should be innovative and creative in the face of global challenges. Since the 1970’s, cities and other public institutions have been subject to many contemporary management models, such as New Public Management which promotes managerial style of management in the public sector and the use of: benchmarking, crowdsourcing, reengineering, controlling, outsourcing, e-governance. An important strategic document related to urban sustainability is the local spatial development plan. Unfortunately, many Polish cities do not have these documents. The average planning coverage is 49.6% for cities (30.2% for Poland), and only 15.6% of areas have project of plans (7.2% for Poland). Additionally planning coverage are characterized by a great diversity, for instance Lodz is covered by plans only 16.1%, but Gdansk—65.4%. In the cities 40.9% of planned local plans have been in preparation for more than three years, which indicates a long process of developing planning documents [23, 24].

**Fig 1:**
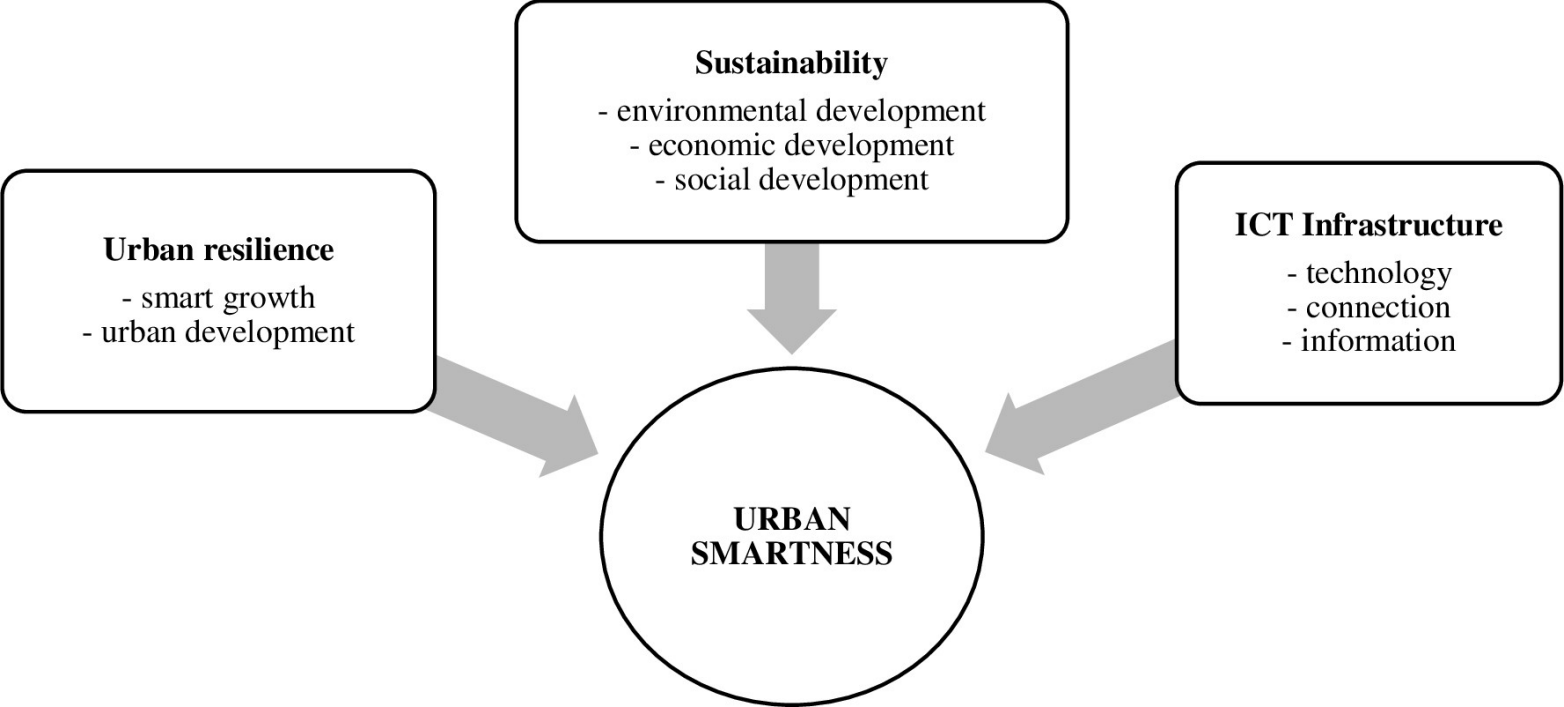
Components of urban smartness (Note: author’s elaboration on the based [21, 25]).

Marsal-Llacuna [25] suggests that smartness means to contribute to sustainable development and resilience. Smartness in the smart city is when the three pillars of sustainability (environmental, economic and social) are safeguarded while urban resilience is being improved by making use of ICT infrastructure ICT. Smartness in the smart city equals urban smartness which is a combination of three components such as: sustainability, urban resilience and ICT infrastructure (Fig 1). The smart city value chain by Dameri is the basis of urban smartness [21]. The smart city value chain obtains: (i) sustainability (carbon neutral, clean air and water); (ii) quality of life (safe, diverse, leisure, convenience); (iii) smart growth (knowledge, innovation, employment, investments). Furthermore Trindade *et al*. analysed scientific studies focusing on both environmental sustainability and smart city concepts to understand relationship between these two [26].

In the 2014, International Organization for Standardization published ISO 37120 norm which helps to measure and compare urban performance in terms of urban service and quality of life. It is a tool for uniform reporting of the state city’s development in 17 thematic groups such as: education, energy, environment, finance, fire & emergency response, governance, health, recreation, safety, shelter, solid water, telecommunication & innovation, transportation, urban planning, wastewater, water & sanitation. Urban leaders can effectively manage and plan the sustainability of their cities [27, 28]. Moreover, Fox [29] introduces the Global City Indicator Ontology which it addresses the problem of how city indicators and their supporting data are to be published on the Semantic Web.

Arroyo-Caňada and Gil-Lafuente [30] suggest that there are significant differences between western and eastern European cities. Additionally Western European cities, particularly those in the Nordic countries, are the best positioned to attract creative IT designers. Researchers explored fuzzy subsets which composed of 29 factors related to the economy, people, governance, mobility, environment, quality of life. The study focuses on 71 European cities using hierarchical cluster analysis. Similarly, Akande *et al*. [31] note that Nordic cities and cities in Western Europe perform better scores than cities in Eastern Europe. Berlin and other Nordic capital cities lead the ranking, while Sofia and Bucharest obtained the lowest rank scores. Furthermore Maltese *et al*. [32] investigated the relation between smartness and energy dimension concerning renewable energy, energy consumption and energy policy. The study refers 103 Italian NUTS3 province capitals using cluster analysis. Researchers identified four cluster labelled competitive cities e.g. Roma, Milano, specializing cities e.g. Palermo, Catania, attractive cities e.g. Bologna, Verona and liveable cities e.g. Rimini, Como. Papa *et al*. [33] examined 13 Italian metropolitan cities between 2006 and 2014 by using the principal component analysis. Researchers suggest that northern cities perform better than southern cities in reducing private transport and increasing the share of sustainable modes of transport such as public transportation, cycling and car sharing. Besides Alonso *et al*. [34] carried out the mobility and environmental evaluation 62 Spanish cities. Researchers claim that the cities better scored are Valencia, Madrid, Barcelona and Sevilla. Jolliffe and Cadima described some variants of principal component analysis and their application [35].

## Materials and methods

**Fig 2:**
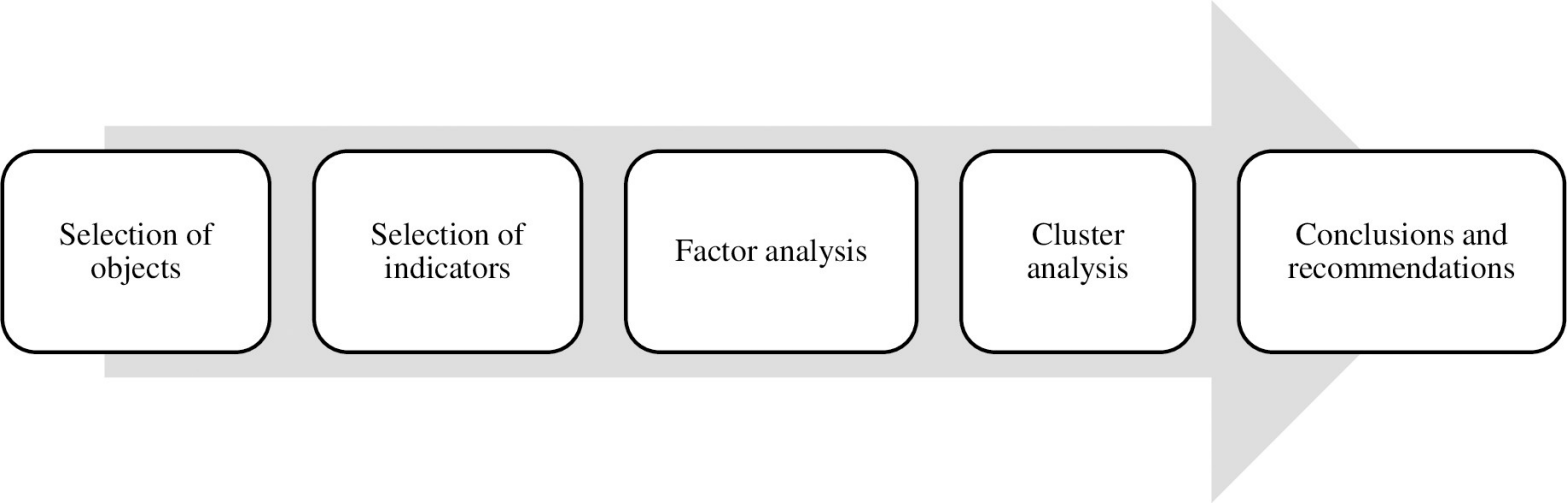
The research design (Note: author’s elaboration).

The test procedure consists of several successive stages: (I) date work (selection of urban sustainability indicators and European cities with ISO37120 standard from the WCCD database; computation of basic statistics; standardization of variables); (II) clustering (estimation of the number of principal factors based on the Kaiser criterion; determining the eigenvalues of the correlation matrix; calculation of the eigenvectors of the correlation matrix; rotation selection; identifying the values of the factor loadings after equamax rotation; calculation of factor scores; for objects; drawing the variables configuration in the two factors space; drawing the objects configuration in the two factors space; determining the clusters number from the agglomeration chart; grouping of cities on the basis of cluster analysis for Ward agglomeration with the Euclidean distance from the link tree diagram; characterization of each cluster based on the k-means analysis from the mean variable graph); (III) results analysis—finding recommendations. The most important stages of the research procedure were visualized in the Fig 2.

The selection of urban smartness indicators is a huge challenge because this issue is approached in the scientific literature, international strategic documents and reports of various organizations in so many different ways. Indicators from the ISO 37120 standard were used in the conducted study. The choice of indicators (structure or intensity) was motivate by suggestions from Transforming our World: The 2030 Agenda for Sustainable Development [36]. The availability of statistical data at the city level becomes the second criterion. Empirical materials within this study were based on currently available statistical data listed by the World Council on City Data between 2014 and 2017. There are 100 indicators of urban service and life quality (46 basic and 54 additional) for 54 cities. The research facilities were selected from the list of cities with the ISO 37120 standard (www.open.dataforcities.org). The following analysis includes only European cities. Table 2 presents the general overview of the analyzed cities.

**Table 2:** The general overview of the analyzed cities.

City	Country	Year of certification ISO 37120	Population	Area [km^2^]	Population density
**Amsterdam**	Netherlands	2014	834,713	164.7	5,065.0
**Eindhoven**	Netherlands	2016	224,788	88.8	2,530.3
**Heerlen**	Netherlands	2016	87,406	45.5	1,944.0
**Rotterdam**	Netherlands	2014	618,357	208.9	2,959.0
**The Hague**	Netherlands	2017	519,988	98.1	5,299.0
**Zwolle**	Netherlands	2017	124,896	119.3	1,046.0
**London**	United Kingdom	2014	**8,538,700**	**1,572.0**	5,341.7
**Koprivnica**	Croatia	2016	30,872	90.9	339.0
**Zagreb**	Croatia	2016	790,017	641.3	1,232.5
**Aalter**	Belgium	2017	*20*,*218*	81.9	*247*.*0*
**Gdynia**	Poland	2017	247,478	135.0	1,831.0
**Kielce**	Poland	2017	197,704	110.0	1,797.3
**Barcelona**	Spain	2014	1,611,822	102.2	**15,777.4**
**Valencia**	Spain	2015	787,266	137.5	5,849.2
**Porto**	Portugal	2016	214,329	*41*.*4*	5,180.5
**Sintra**	Portugal	2017	382,521	319.2	1,198.3

A italic font–a minimum value; a bold font–a maximum value.

Note: author’s elaboration on the based WCCD ISO37120.

**Fig 3:**
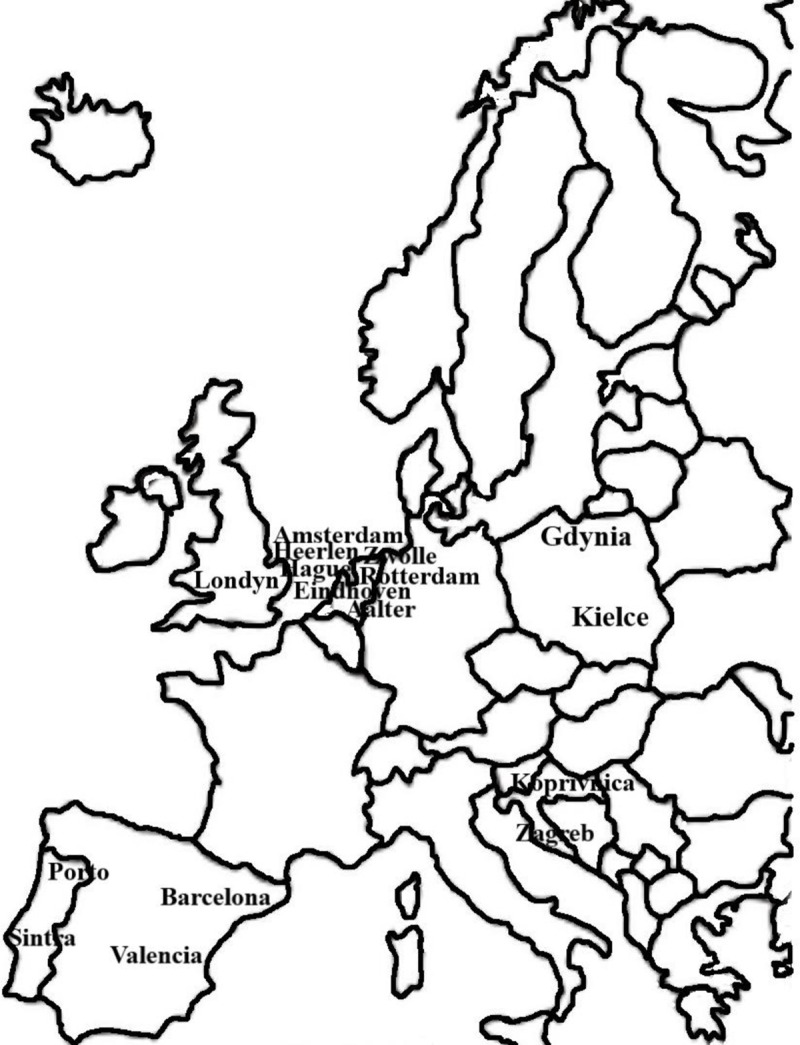
The location of the analyzed cities (Note: author’s elaboration).

The selection of analyzed cities was carried out using three criteria: (i) spatial coverage concerns Europe; (ii) possession of an ISO 37120 certificate at the platinum level; (iii) all mandatory indicators identified. Fig 3 shows the location of the analyzed cities.

The selection of diagnostic variables to assess sustainable urban development included several stages. It was checked whether the variables fulfill the formal criteria in terms of measurability, completeness and comparability. Regarding statistical premises, the set of variables eliminated those for which the coefficient of variation was below 10%. A further reduction of the variables related to excessive correlation with the analysis of the matrix of coefficients used Pearson correlations. It allowed the identification of diagnostic features that were excessively correlated, which should be removed from further research. Finally, the following indicators were selected for analysis: a share of city's unemployment, a ratio of primary education student to teacher, a amount of fine particulate matter (PM2,5) concentration, a number of firefighters per 100,000 population, a number of total collected municipal solid waste per capita, a number of green area per 100,000 population.

The evaluation of sustainable development is very complex due to the wide range of factors. Assessment of urban sustainability requires the determination of a set of indicators characterizing key aspects in three dimensions economic (X_1_), social (X_2_, X_4_) and environmental (X_3_, X_5_, X_6_) as well as an indication of their importance for sustainable development. The research was carried out using STATISTICA version 13.1 program and EXCEL. Table 3 presents the list of analyzed cities with the characteristics of sustainability.

**Table 3:** The characteristics of indicators in analyzed cities.

City	X_1_	X_2_	X_3_	X_4_	X_5_	X_6_
	%	-	μg/m^3^	units/ 100,000	t/capita	hectares/100,000
**Amsterdam**	7.6	16.7	13.6	61.2	0.37	595.4
**Eindhoven**	8.3	17.5	15.1	38.0	0.50	869.0
**Heerlen**	8.6	19.3	15.2	37.4	0.56	995.0
**Rotterdam**	12.6	14.7	14.9	34.6	0.43	652.0
**The Hague**	8,8	16.7	11.9	98.3	0.49	472.0
**Zwolle**	7.0	19.5	10.4	32.0	0.54	886,0
**London**	7.2	18.9	14.6	63.8	0.42	338.9
**Koprivnica**	10.4	13.0	15.5	**220.3**	0.29	140.0
**Zagreb**	9.6	*11*.*5*	**21.0**	46.1	0.39	83.9
**Aalter**	*3*.*3*	18.2	12.2	*9*.*9*	*0*.*13*	**4,465.8**
**Gdynia**	4.9	13.2	13.5	130.0	0.36	347.4
**Kielce**	7,7	12.2	20.7	102.7	0.33	464.9
**Barcelona**	17.0	**22.7**	15.3	36.1	0.45	180.6
**Valencia**	**21.7**	21.4	12.0	36.2	0.39	*46*.*9*
**Porto**	17.6	13.1	*5*.*1*	99.8	**0.63**	133.2
**Sintra**	6.3	12.4	12.2	43.1	0.40	192.1

A italic font–a minimum value; a bold font–a maximum value

(X_1_) a share of city's unemployment; (X_2_) a ratio of primary education student to teacher; (X_3_) a amount of fine particulate matter (PM2,5) concentration; (X_4_) a number of firefighters per 100,000 population; (X_5_) a number of total collected municipal solid waste per capita; (X_6_) a number of green areas per 100,000 population.

Note: author’s elaboration on the based WCCD ISO37120.

The factor analysis was developed by C. Spearman in 1904 [37]. It is a popular multivariate method used for data reduction purpose. The basic idea is to represent a set of variables by a smaller number of factors. The variables used in factor analysis should be linearly related to each other. This can be checked by looking at scatterplots of pairs of variables.

Thus, the factor analysis model can be expressed using the following formula:

$$X_{i}=a_{i1}F_{1}+a_{i2}F_{2}+\cdots +a_{im}F_{m}+e_{i}$$ (1)

where: *X*_1_,*X*_2_,…,*X*_*p*_—*p* variables, then variable *i* can be written as a linear combination of *m* factors *F*_1_,*F*_2_,…,*F*_*m*_, *m*<*p*;

*a*_*i*_—the factor loadings for variable *i*;

*e*_*i*_—the part of variable *X*_*i*_.

The cluster analysis, derived from the modeling classification, was developed by R.C. Tryon in 1939 [38]. It is a popular a method of grouping a set of objects in such a way that objects in the same group are more similar to each other than to those in other groups. The cluster analysis refers to data mining and machine learning. Grouping is strictly conditioned by the data source and the expected form of results. The cluster analysis algorithms are divided into two basic categories of hierarchical and non-hierarchical methods. Agglomerative procedures create a similarity matrix of classified objects, and then in the next steps combine the most similar objects into clusters. K-means methods consists in pre-dividing the set into a predetermined number of classes. The most popular distance is the Euclidean metric, which can be calculated using the following formula:

$$d(p,\;q)=d(q,\;p)=\sqrt{{\left(q_{1}-p_{1}\right)}^{2}+{\left(q_{2}-p_{2}\right)}^{2}+\cdots +{\left(q_{n}-p_{n}\right)}^{2}}=\sqrt{\sum_{i=1}^{n}{\left(q_{i}-p_{i}\right)}^{2}}$$ (2)

where: *p* = (*p*_1_,*p*_2_,…,*p*_*n*_), *q* = (*q*_1_,*q*_2_,…,*q*_*n*_) – two points in the Euclidean n-space,

d–the distance from a point p to a point q.

## Results

The research began with computing the basic statistics for urban indicators by measuring position (arithmetic mean) and variability (standard deviation, variation coefficient, skewness, kurtosis). The most diverse indicator is number of a green areas, while the least is a ratio of primary education student to teacher. Table 4 presents information on general statistics for each indicator. Afterwards indictors were standardized using the following formula:

$$z=\frac{x-\bar{x}}{S_{X}}$$ (3)

**Table 4:** The basic statistics of indicators.

	*M*	*Min*	*Max*	*S*	*V*	*A*	*K*
X_1_	9.92	3.33 Aalter	21.70 Valencia	4.96	49.98	1.206090	0.94073
X_2_	16.32	11.52 Zagreb	22.71 Barcelona	3.53	21.65	0.230733	-1.16097
X_3_	13.95	5.10 Porto	21.00 Zagreb	3.74	26.82	-0.131652	1.86634
X_4_	68.09	9.87 Aalter	220.26 Koprivnica	52.26	76.75	1.831362	3.94211
X_5_	0.42	0.13 Aalter	0.63 Porto	0.12	28.12	-0.558799	1.47621
X_6_	678.94	46.92 Valencia	4,465.80 Aalter	1,054.14	155.26	3.462621	12.93413

*M*—arithmetic mean; *Min*—minimum value; *Max*—maximum value; *S*—standard deviation; *V*—coefficient of variation; *A*—skewness; *K*- kurtosis.

Note: author’s elaboration on the basis of STATISTICA 13.1.

The next stage was to determine the eigenvalues of the correlation matrix (Table 5). The eigenvalues for a given factor measure the variance in all the variables which is accounted for by that factor. The ratio of eigenvalues is the ratio of explanatory importance of the factors with respect to the variables. If a factor has a low eigenvalue, then it is contributing little to the explanation of variances in the variables and may be ignored as redundant as compared to more important factors. It reflects the significance of factors in explaining the information of input variables (percentage in the variability of the data set). The number of factors was determined using the eigenvalues method greater than 1 (a Kaiser criterion). The decision on the number of factors can also be made on the basic of the scree criterion. The higher the correlation coefficient of a variable with a factor means the higher the significance of the variable for a given factor.

**Table 5:** The eigenvalues of the correlation matrix.

Value number	Eigenvalues	Variance [%]	Cumulative eigenvalues	Cumulative Variance [%]
**1**	**1.844699**	**42.56670**	**1.844699**	**42.56670**
**2**	**1.497700**	**34.55966**	**3.342399**	**77.12636**
3	0.588016	13.56855	3.930414	90.69490
4	0.393098	9.07080	4.323512	99.76570
5	0.005189	0.11973	4.328701	99.88543
6	0.004965	0.11457	4.333666	100.00000

Note: author’s elaboration on the basis of STATISTICA 13.1.

There are several ways to conduct factor analysis for instance unweighted least squares, generalized least squares, maximum likelihood. The interpretability of factors improved through rotation. There are many different types of rotation, but they try make factors each highly responsive to a small subset of items. Rotation works through changing the absolute values of the variables whilst keeping their differential values constant. There are two major categories of rotations as orthogonal and oblique. Orthogonal rotations produce uncorrelated factors, but oblique—correlated factors. Varimax, quartimax and equamax are the variant of orthogonal rotation. The most commonly oblique rotations are Direct Quartimin, Promax and Harris-Kaiser Orthoblique.

The next step of investigation was to determine the values of the factor loadings after equamax rotation (Table 6). Each of measures are linearly related to each factors. The strength of this relationship is contained in the respective factor loading, produced by rotation. This loading is interpreted as a standardized regression coefficient, regressing the factor on the measure.

**Table 6:** The values of the factor loadings after equamax rotation.

Variables	Factor 1	Factor 2
X_1_	**-0.624872**	0.024753
X_2_	-0.480099	**0.885440**
X_3_	0.282024	-0.067826
X_4_	0.092310	**-0.510658**
X_5_	**-0.728974**	0.014362
X_6_	**0.647019**	**0.855507**

Note: author’s elaboration on the basis of STATISTICA 13.1.

Consequently, it was calculated the projection of each observation on each of the factor. The factor scores gave the location of each observation in the space of the common factors. Table 7 presents the factor scores for objects.

**Table 7:** The factor scores for objects.

	Amsterdam	Eindhoven	Heerlen	Rotterdam	Hague	Zwolle	London	Koprivnica	Zagreb	Aalter	Gdynia	Kielce	Barcelona	Valencia	Porto	Sintra
Factor 1	-0.00085	-0.19009	-0.49659	0.18155	-0.37554	-0.71328	-0.53364	0.57077	0.65316	2.54653	0.45071	0.94909	-1.34059	-1.28439	-0.67674	0.25989
Factor 2	-0.93388	1.08908	2.29738	-0.21264	0.23991	0.66529	-0.26491	-0.54089	-1.06215	2.59781	-1.56048	-0.34347	1.22611	-0.11400	-0.31370	-2.76948

Note: author’s elaboration on the basis of STATISTICA 13.1.

**Fig 4:**
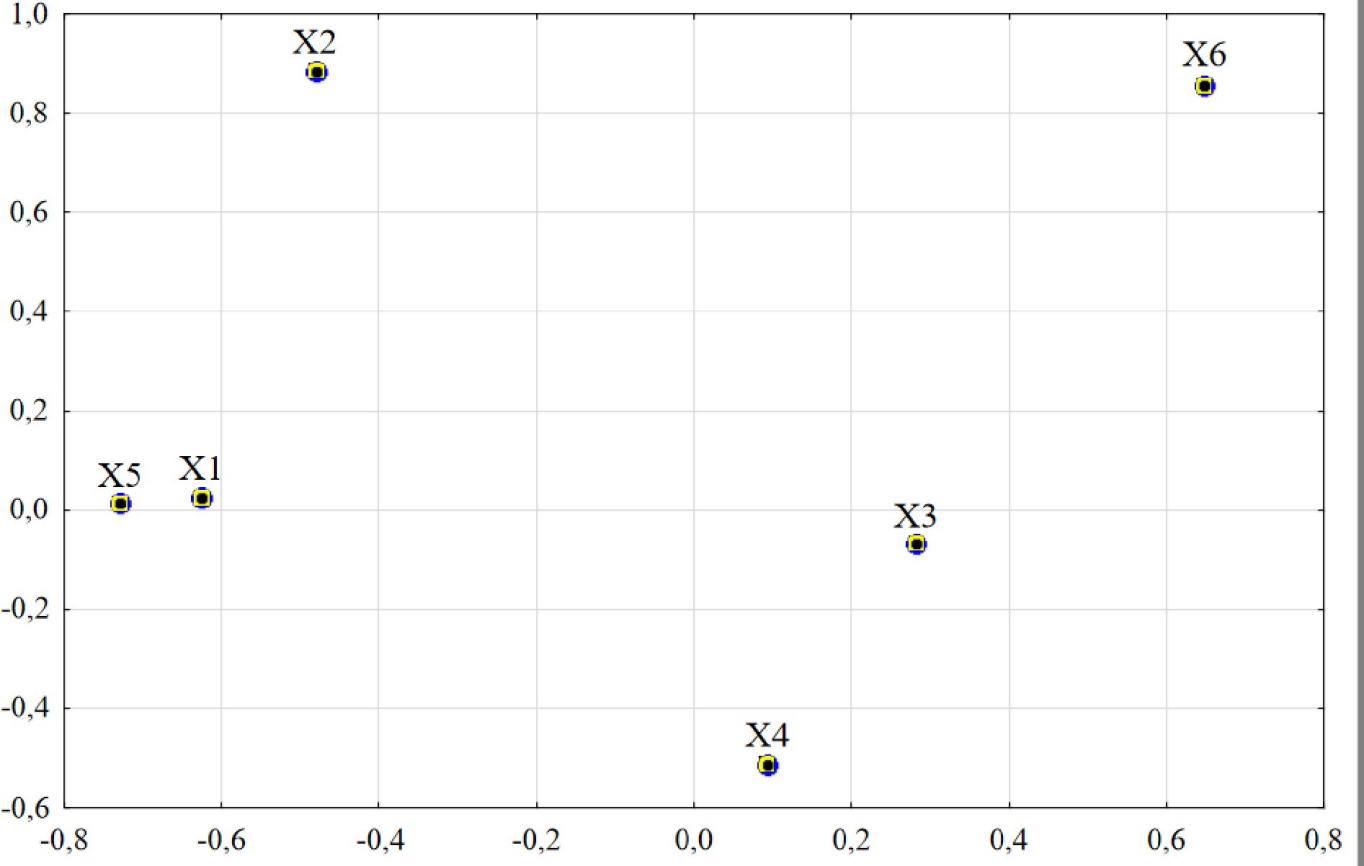
The variables configuration in the two factors space (Note: author’s elaboration on the basis of STATISTICA 13.1).

The Fig 4 shows graphically the dependence between input variables and the obtained factors.

**Fig 5:**
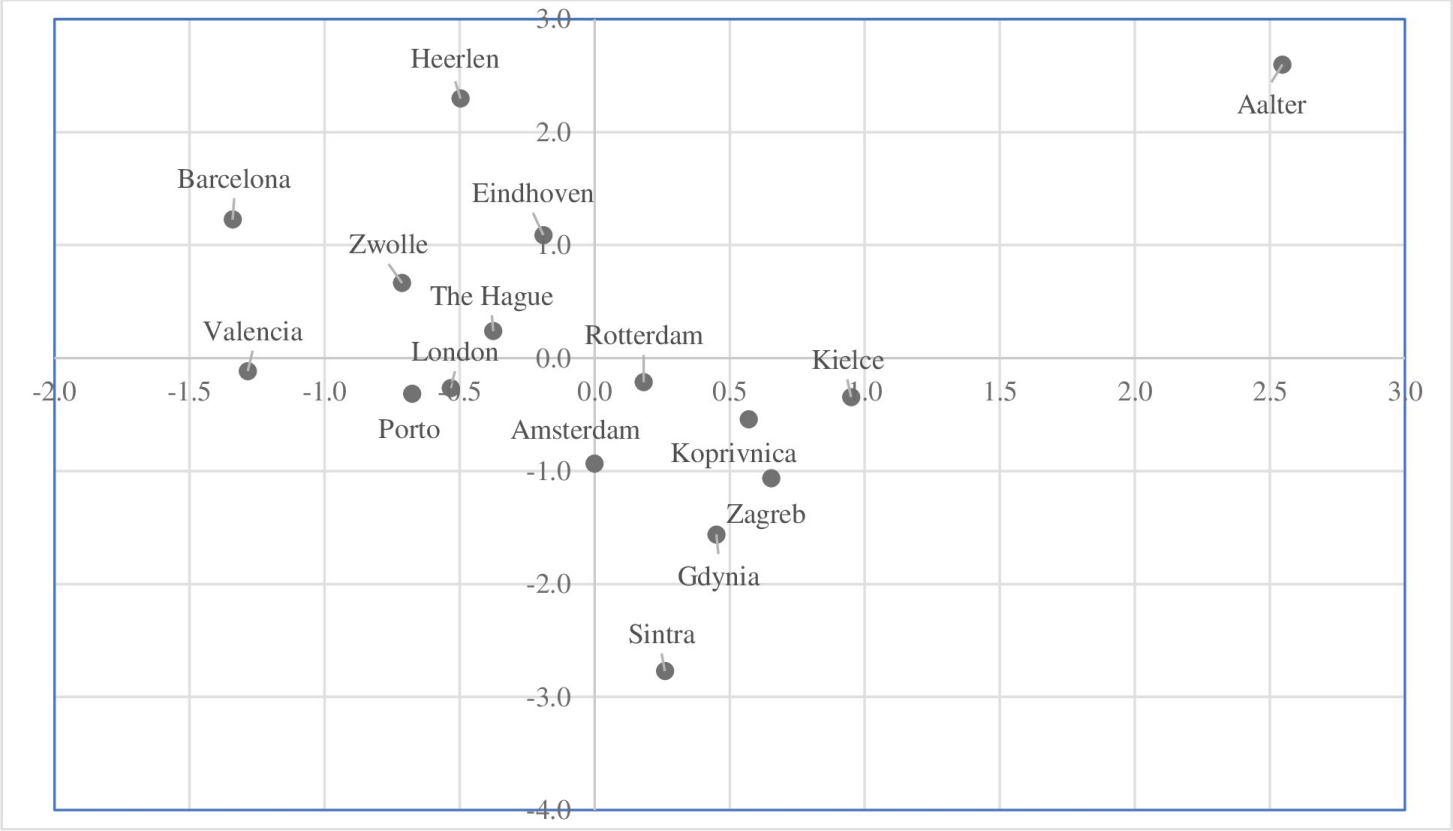
The objects configuration in the two factors space (Note: author’s elaboration on the basis of STATISTICA 13.1).

The next step of the study involved the identification of outliers based on the configuration of objects in the space of the two factors space. The Fig 5 presents the graphic location of cities in the two factors space.

**Fig 6:**
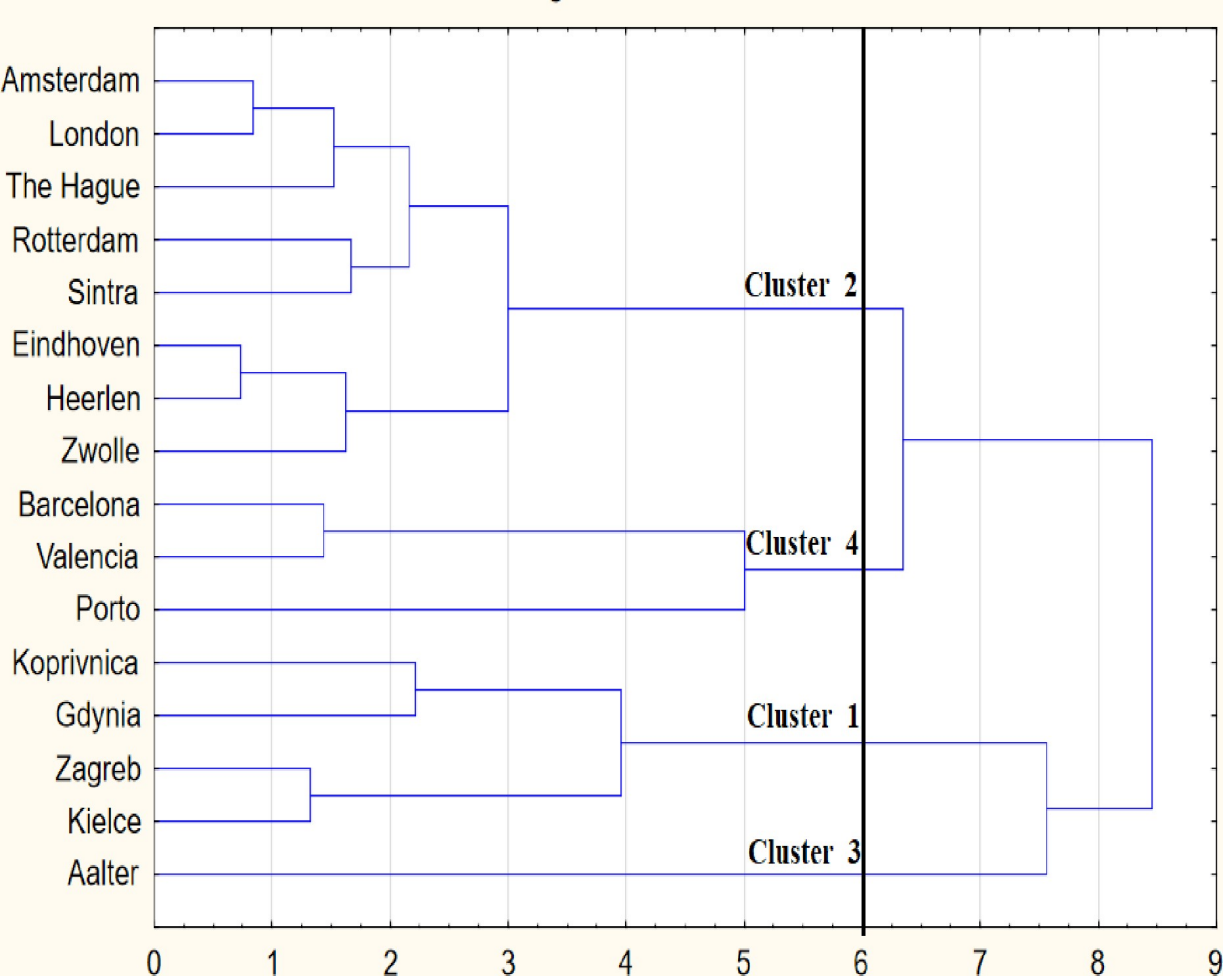
The dendrogram and clusters (Note: author’s elaboration on the basis of STATISTICA 13.1).

**Fig 7:**
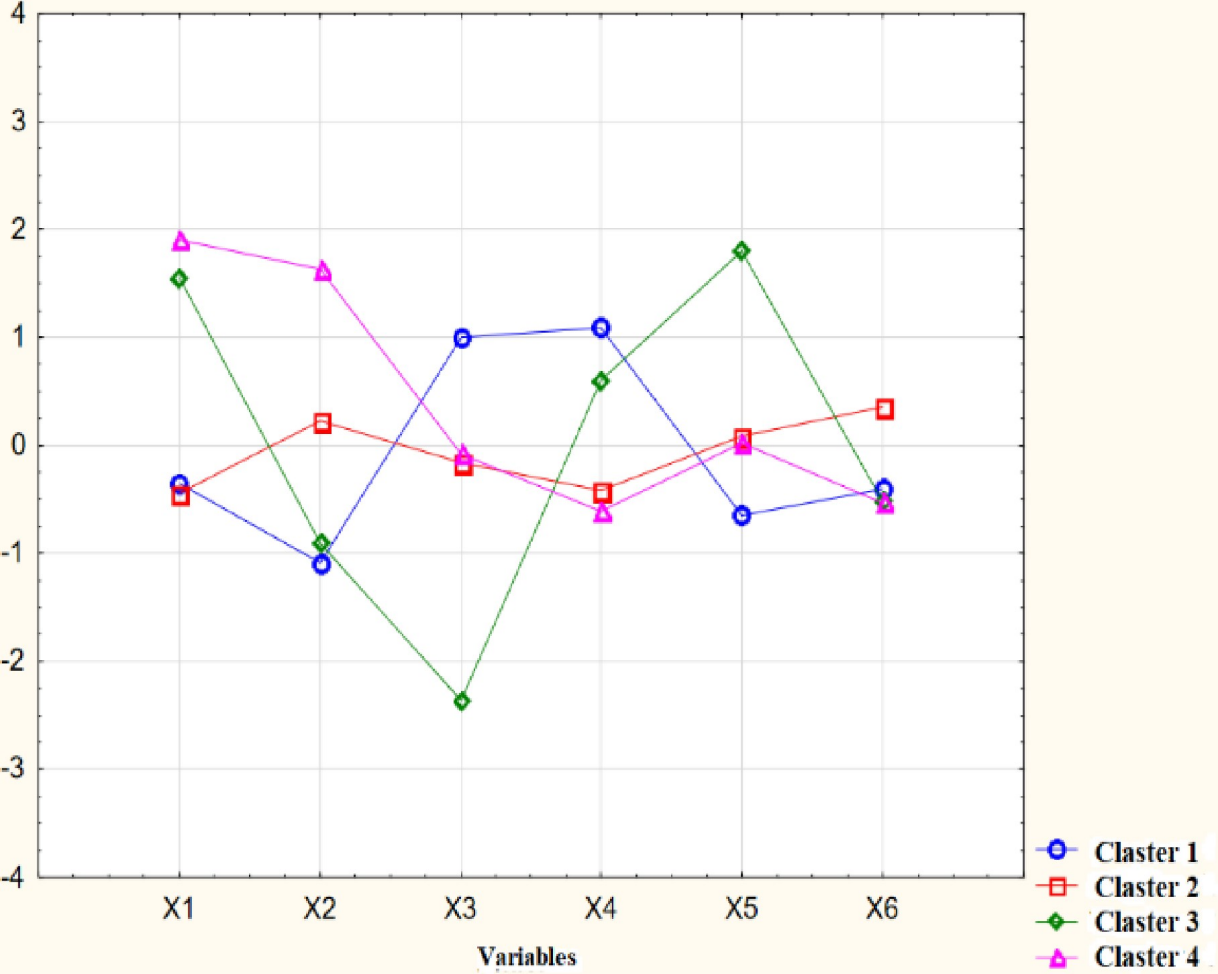
The graph of variables’ average values in clusters (Note: author’s elaboration on the basis of STATISTICA 13.1).

The next step involved the cluster analysis. Grouping was carried out using agglomeration and k-means methods. In the agglomeration analysis, the Ward method was selected, in which the Euclidean distance was used to compare cities. The agglomeration graph presents information about the binding distance relative to the binding steps. The objects clusters have been identified in the dendrogram—sopel chart (Fig 6). Groups of objects were characterized by the k-means cluster analysis. The graph of variables’ average values in individual clusters contains information about the best and the worst cluster of cities (Fig 7).

Table 8 presents the assessment of the level of sustainable and smart development based on the average values of indicators for individual city clusters.

**Table 8:** The indicators’ average value in individual clusters.

The cluster	Cities	X_1_	X_2_	X_3_	X_4_	X_5_	X_6_
**Cluster 1**	Gdynia, Kielce, Koprivnica, Zagreb	8.1	*12*.*5*	**17.7**	**124.8**	0.34	259.05
**Cluster 2**	Amsterdam, Eindhoven, Heerlen London, Rotterdam, Sintra, The Hague, Zwolle	8.3	17.0	13.5	51.1	0.47	625.05
**Cluster 3**	Aalter	*3*.*3*	18.2	12.2	*9*.*9*	*0*.*13*	**4,465.8**
**Cluster 4**	Barcelona, Porto, Valencia	**18.8**	**19.1**	*10*.*8*	57.4	**0.49**	*120*.*24*

A italic font–a minimum value; a bold font–a maximum value.

Note: author’s elaboration on the basis of STATISTICA 13.1.

## Discussion

The considerations, carried out in the manuscript, allowed to state that the smart city concept is implemented using sustainability in economic, social and environmental aspects. The application of the factor analysis presented relationships between indicators characterizing sustainability in selected European cities. The evaluation of the implementation of sustainability using cluster methods has allowed to identify similar cities.

In the case being analyzed, the impact of input indicators on the sustainability of cities was described through the first two factors (Table 5). The two factors contain 77.13% of the variability of input variables. The first factor transfers 42.57% of the information contained in the input variables. The second factor explains 34.56% of the variability of the input data.

The first factor consists of positively correlated variables (Table 6) with a green area (X_6_) as well as those that are negatively correlated: a total collected municipal solid waste (X_5_) and a city's unemployment (X_1_). The above correlations testify to the association of high values of variables X_5_, X_1_ with low value of the number of firefighters (X_4_) and accordingly, with the increase of the latter the former decrease. The second factor consists of positively correlated variables with a ratio of primary education student to teacher (X_2_) and a green area (X_6_) as well as also negatively with a responsible for number of firefighters (X_4_).

In the analyzed example, most of the information contained in input variables is transferred by the factors (Fig 4). The strong correlation (two variables next to each other) occurs between a city's unemployment (X_1_) and a collected municipal solid waste (X_5_). The lack of correlation is between a fine particulate matter concentration (X_3_) and a green area (X_6_) or a collected municipal solid waste (X_5_) and a ratio of primary education student to teacher (X_2_). The negatively correlated is between a number of firefighters (X_4_) related to a ratio of primary education student to teacher (X_2_).

The position of cities shows the graph of objects’ configuration in the two factors space (Fig 5). Aalter (cluster 3) is outlying city because of the high level of socio-economic development. Koprivnica (cluster 1) and Porto (cluster 4) are distinctive from the other cities. The results of grouping cities by methods of the cluster analysis (Fig 6) and the factor analysis (Fig 5) are identical.

The level identifying the cluster number at 13th step corresponds to 6 binding distances On the basis of the agglomeration graph,. The four clusters of cities were identified on the dendrogram (Fig 6). The urban indicators were characterized using the k-means analysis. The graph of variables’ average values in particular clusters presents information about the best and the worst group of cities. The fourth cluster is the weakest set of cities, but the third cluster is the best set of cities.

The clusters’ characteristics were prepared based on the k-means analysis (Fig 7).

The cluster 2 is the most numerous group. It consists of eight cities (Amsterdam, Eindhoven, Heerlen London, Rotterdam, Sintra, The Hague, Zwolle). The number of a green areas is higher (625.05 hectares/100,000), and the remaining indicators’ average values have an medium value.

The cluster 3 is the least numerous group with an isolated Aalter. The value of a green areas is the highest (4,465.8 hectares/100,000) as well as a unemployment (3.3%), the number of firefighters (9.9 units/100,000) and the amount of collected municipal solid waste (0.13 t/capita) are the lowest.

The cluster 4 consists of three cities (Barcelona, Porto, Valencia). The value of a unemployment (18.8%), the ratio of primary education students to teachers (19.1) and the amount of collected municipal solid waste (0.49 t/capita) are the highest. The amount of fine particulate matter concentration (10.8 μg/m^3^) and a green areas (120.24 hectares/100,000) are the lowest.

Finally, cluster 1 includes four cities (Gdynia, Kielce, Koprivnica, Zagreb). The amount of fine particulate matter concentration (17.7 μg/m^3^) and the number of firefighters (124.8 units/100,000) are the highest. The ratio of primary education student to teacher (12.5) is the lowest. Table 9 shows the assessment of urban smartness.

**Table 9:** Assessment of urban smartness.

The cluster	The main pillars of smart cities			The level of urban smartness
	Economic	Social	Environmental	
**Cluster 1**	+/-	++	+/-	Average smartness
**Cluster 2**	+	+	+	Higher smartness
**Cluster 3**	++	+/-	++	The highest smartness
**Cluster 4**	-	+	-	Low smartness

(++) strong relation; (+) positive relation; (+/-) neutral relation; (-) negative relation.

Note: author’s elaboration.

If we look at scientific literature, the factor analysis is rarely used to study the diversity of indicators of sustainability in cities. Salvati [39] applied this method to identify factors shaping land consumption in 155 European cities. The Northern European and United Kingdom cities had the lowest level of land consumption. Furthermore Yan *et al*. [40] assessed the performance of urban sustainability in Chinese cities based on natural resource input (water, energy, land) and human welfare (safety, health, basic material for good life, freedom of choice, freedom of action) using data envelopment analysis. An interesting investigation was conducted by Gonzalez-Garcia [41]. Spanish cities evaluated on the basis of ratio of people at risk of poverty and social exclusion, the unemployment rate, criminology ratio, educational places, education level, net disposable income as well as an environmental endpoint.

## Conclusions

This paper indicates the possibility of using a certain methodology to study the urban smartness. The smart city is rapidly becoming a key success factor for contemporary urban world. The importance of the smart city concept has increased along with the development of the globalization process. Given these facts, the paper aim was to assess of the urban smartness of selected European cities. The presentation of the unique quantitative research related to this topic can be regarded as evidence of the originality of the manuscript. Using the analysis of selected European cities data, the findings brought diversified results, allowing to answer the research questions. This study contributes to the knowledge base in several ways. Firstly, thought this research adopts a single-continent approach, analyzing smart cities in Europe gives the opportunity to compare the results with other continents. Secondly, given the growing role of the smart city concept, it is expected that many decision makers would have to take this growing trend into account if they wish to help achieve sustainability in urban development. The result of this study can offer guidance for city managers willing to obtain benefits from the implication of the smart city concept. This study had several limitations, the most important of which was the analysis of only a few variables and selected cities.

In this study cluster analysis and factor analysis were used to identify significantly variables related to sustainability of selected European cities with the implemented ISO 37120 standard. The method allows replacing the input set of correlated features with a small number of uncorrelated factor which are linear combinations of variables. Two of the extracting factors explain nearly 77% the variability of input data. The first factor justifies 43% of the variability of input data. The second factor explains almost 34% of the variability of data. It can be concluded that the factor analysis is useful in reducing the dimensionality of variables in the description of the problem under considered. The first factor mainly contains the a green area (X_6_), but the second factor—a ratio of primary education student to teacher (X_2_). The strong correlation occurs between the city's unemployment (X_1_) and the collected municipal solid waste (X_5_). Aalter, Koprivnica and Porto are distinctive from the other cities. The cluster analysis allowed to identify and characterize four groups of similar cities. The fourth cluster (Barcelona, Porto, Valencia) is the weakest set of cities, but the third cluster (only Aalter) is the best set of cities. The results of grouping cities by methods of the cluster analysis and the factor analysis are identical. The conducted research shows that the analysed cities present a huge diversity of the sustainability level.

This ranking is intended to attract attention and induce competition between cities. The city managers can see in the objective state of the extent in which they are perceived as smart and sustainable, but also are able to identify the points in which to improve their sustainability. The proposed test procedure can be used to assess the sustainability level of other European and non-European cities.

Aalter (cluster 3) has the highest level of urban smartness because of the environmental and the economic pillars of sustainability. The main pillar of sustainability is social in the cluster 1. The cluster 4 presents the low urban smartness because of the environmental and the economic pillars of sustainability.

The paper fills the gap in the editorial market by reviewing issues related to urban smartness through the use of extensive literature. Another advantage of the article is the application of an original cluster and factor analysis methods to assess the indicators of cities in the area of urban smartness. The further research could be conducted through direct interviews with city managers and players in order to understand their attitude towards the development of smart city projects. However, the research carried out in this manuscript does not fully cover the extensive research topic. Interesting research on the future of urban smartness should include following issues: (i) operationalization of urban smartness measurement; (ii) determinants of the level of urban smartness depending on the size and type of urban units; (iii) conceptualization of the urban smartness model; (iv) using e-Planning tools to build a smart city strategy.

## References

[1] United Nations. The World’s Cities in 2016. Available online: http://www.un.org/en/development/desa/population/publications. Accessed on 08.02.2020.

[2] European Union. Mapping Smart Cities in the EU 2014. Available online: www.europarl.europa.eu/ (accessed on 02.01.2020).

[3] Fernandez-Anez, V. Stakeholders Approach to Smart Cities: A Survey on Smart City Definitions. In *Smart Cities. Smart-CT 2016. Lecture in Computer Science*, Alba E., Chicano F., Luque G., Eds., Proceedings of the First International Conference on Smart Cities. Smart-CT 2016, Malaga, Spain, 15–17 June 2016; E., Alba, F. Chicano, G., Luque, Eds., Springer: Cham, Switzerland, 2016, Vol. 9704, 157–167; 10.1007/978-3-319-39595-1_16

[4] Hollands R. Will the real smart city please stand up?. *City*, 2008, 12(3), 303–320; 10.1080/13604810802479126

[5] Harrison C.; Eckman B.; Hamilton R.; Hartswick P.; Kalagnanam J.; Paraszczak J.; et al Foundations for Smarter Cities. *IBM Journal of Research and Development*, 2010, 54(4), 1–16, 10.1147/jrd.2010.2048257

[6] Nam, T.; Pardo, T.A. Conceptualizing Smart City with Dimensions of Technology, people, and institutions. In Digital Government Research, Proceedings Paper of the 12^th^ Annual International *Digital Government Research* Conference on Digital Government Innovation in Challenging Times, College Park, Maryland, USA, June 12–15 2011; John Bertot, Karine Nahon, Eds.; ACM: New York, USA, 2011, 282–291, 10.1145/2037556.2037602

[7] Lombardi P.; Giordano S.; Farouh H.; Yousef W. Modelling the smart city performance. *Innovation*: *The European Journal of Social Science Research*, 2012, 25(2), 137–149; 10.1080/13511610.2012.66032

[8] Giffinger R., Fertner C., Kramar H., Kalasek R., Pichler-Milanoić N., Meijers E. *Smart cities*. *Ranking of European medium-size cities*, Centrel of Regional Science, University of Technology, Vienna 2007 Available online: http:///www.smart-cities.eu/download/smart_cities_final_report.pdf (accessed on 07.09.2018).

[9] Cocchi A. Smart and digital city: A systematic literature review In *Smart city*. *How to Create Public and Economic Value with High Technology in Urban Space* Dameri, Rosenthal-Sabroux R.P.,, C., Eds.; Springer: Cham, Switzerland, 2014, pp. 13–43; ISBN 978-3-319-06159-7.

[10] Clarke R.Y. Measuring Success in the Development of Smart and Sustainable Cities. In *Managing for Social Impact*. *Innovations in Responsible Enterprise*. *Management for Profesional*, Cronin M.J.,, Dearing T.C., Eds.; Springer: Cham, Switzerland, 2017; pp. 239–254; ISBN 978-3-319-46020-8.

[11] Dameri R.P. Searching for smart city definition: a comprehensive proposal, *International Journal of Computers & Technology*, 2013, 11(5), 2544–2551; 10.24297/ijct.v11i5.1142

[12] Van der Meer A.; Van Vinden W. E-governance in Cities: A Comparison of Urban Information and Communication Technology Policies, *Regional Studies*, 2003, 37(4), 407–419; 10.1080/0034340032000074433

[13] Ramaprasad, A.; Sanchez-Ortiz, A.; Syn, T. A Unified of a Smart City. In *Electronic Government and the Information Systems Perspective. Lecture Notes in Computer Science*, Proceedings of the 6^th^ International Conference on Electronic Government and the Information Systems Perspective EGOVIS 2017, Lyon, France, 28–31 August 2017; A., Kö, E. Francesconi, Eds., Springer: Cham, Switzerland, 2017, Vol. 10441, 13–24; 10.1007/978-3-319-64677-0_2

[14] Ferrara R. The Smart city and the Green Economy in Europe: A Critical Approach, *Energies*, 2015, 8(6), 4724–4734; 10.3390/en8064724

[15] Thuzar M. Urbanization in SouthEast Asia: Developing Smart Cities for the Future? In *Regional Outlook*: *Southeast Asia 2011–2012*; Montesano M.J., Lee P.O., Eds.; ISEAS-Yusof Ishak Institute Singapore: Singapore 2011, pp. 96–100; ISBN 9789814311694.

[16] Caragliu A.; del Bo C.; Nijkamp P. Smart cities in Europe. *Journal of Urban Technology*, 2011, 18(2), 65–82; 10.1080/10630732.2011.601117

[17] Barrionuevo J.M.; Berrone P.; Ricart J.E. Smart Cities, Sustainable Progress: Opportunities for Urban Development. *IESE Insight*, 2012, 14, 50–57; 50–57 10.15581/002.art-2152

[18] Lazaroiu G.C., Roscia M. Definition Methodology for the Smart Cities Model. *Energy* 2012, 47(1), 326–332; 10.1016/j.energy.2012.09.028

[19] Bakici T., Almirall E., Wareham J. A Smart City Initiative: The Case of Barcelona. *Journal of the Knowledge Economy*, 2012, 4(2), 135–148; 10.1007/s13132-012-0084-9

[20] Zygiaris S. Smart City Reference Model: Assisting Planners to Conceptualize the Building of Smart City Innovation Ecosystems. *Journal of the Knowledge Economy*, 2013, 4(2), 217–231; 10.1007/s13132-012-0089-4

[21] Dameri R.P., Urban Smart Dashboard Measuring Smart City Performance In *Smart City Implementation*. *Creating Economic and Public Value in Innovation Urban Systems*, Dameri P., Springer, Cham, 2017, 67–84. 2073/10.1007/978-3-319-45766-6_4. 10.1093/imaman/dpw024

[22] Wiseman J.; Edwards T.; Luckins K. Pathway to a sustainable and resilient urban future: economic paradigm shifts and policy priorities In *Resilient Sustainable Cities*: *a Future*; Pearson L., Newton P., Roberts P. Eds.; Routledge: Abingdon, Oxon, 2014, pp. 31–44. ISBN. 978-0-415-81621-2.

[23] Hajduk, S. Selected Aspects of Measuring Performance of Smart Cities in Spatial Management. In *Business and Management-Spausdinta*, Proceedings Paper of 9^rd^ International Scientific Conference on Business and Management, Vilnius, Lithuania, May 12–13 2016; Stankeviciene J., Lankauskiene T. Eds.; 10.3846/bm.2016.57

[24] Hajduk S., Instruments of Spatial Management in the Context of Sustainability–a Multi-Dimensional Comparative Analysis of the Regional Cities, *Annual Set The Environment Protection* 2018, T. 20, s. 1219–1233, ISSN 1506-218X.

[25] Marsal-Llacuna, M.L. Measuring the Standardized Definition of “smart city”: A Proposal on Global Metrics to Set the Terms of Reference for Urban “smartness”. In *Computational Science and Its Applications. Lecture Notes in Computer Science*, Proceedings of the 15^th^ International Conference on Computational Science and Its Applications–ICCSA 2015, Banff, AB, Canada, 22–25 June 2015; O. Gervasi, B. Murante, S. Misra, M.L. Gavrilova, A.M. Rocha, C. Torre, D. Taniar, B.O. Apduhan, Eds.; Springer: Cham, Switzerland, 2015, vol. 9155, 593–611; 10.1007/978-3-319-21407-8_42

[26] Trindade E.P., Hinnig M.P.F., da Costa E.M. et al Sustainable development of smart cities: a systematic review of the literature. J. open innov. 3, 11 (2017). 10.1186/s40852-017-0063-2.

[27] Fox, M.S.; Pettit, Ch.J. On the completeness of open city data for measuring city indicators. In *International Smart Cities Conference*, Proceeding of the 2015 IEEE First International Smart Cities Conference (ISC2), Guadalajara, Mexico, 25–28 October 2015; IEEE; 10.1109/ISC2.2015.7366147

[28] McCarney P. (2015). The evolution of global city indicators and ISO 37120: The first international standard on city indicators. *Statistical Journal of the IAOS*, 31, 103–110.

[29] Fox M.S. The role of ontologies in publishing and analysing city indicators. *Computers*, *Environment and Urban Systems*, 2015, 54, 266–279; 10.1016/j.compenvurbsys.2015.09.009

[30] Arroyo-Caňada F.J.; Gil-Lafuente J. Multidimensional Positioning of a Set of European Smart Cities In *Sustainable Smart Cities*. *Innovation*, *Technology*, *and Knowledge Management*; Peris-Ortiz M., Ed.; Springer: Cham, Switzerland, 2017, pp. 49–63. ISBN. 978-3-319-40894-1.

[31] Akande A.; Cabral P.; Gomes P.; Casteleyn S. The Lisbon ranking for smart sustainable cities in Europe. *Sustainable Cities and Society*, 2019, 44, 475–487; 10.1016/j.scs.2018.10.009

[32] Maltese I.; Mariotti I.; Boscacci F. Smart City, Urban Performance and Energy In *Smart Energy in the Smart City*. *Green Energy and Technology*. *Urban Planning for a Sustainable Future*; Papa R., Fistola R, Eds.; Springer: Cham, Switzerland 2016, pp. 25–42. ISBN 978-3-319-31155-5.

[33] Papa, R.; Gargiulo, C.; Russo, L. The evolution of smart mobility strategies and behaviours to build the smart city. In *Models and Technologies for Intelligent Transportation Systems*, Proceedings Paper of the 5^th^ IEEE International Conference on Models and Technologies for Intelligent Transportation Systems MT-ITS, Naples, Italy, 26–28 June 2017, 409–414. 10.1109/mtits.2017.8005707

[34] Aleta, N.B.; Alonso, C.M.; Ruiz, R.M.A. Smart mobility and smart environment in the Spanish cities. In *Transport Research Procedia 24*, Proceedings Paper of the 3^rd^ Conference on Sustainable Urban Mobility CSUM2016, Volos, Greece, 26–27 May 2016, E.G. Nathanail, M.A. Gogas, Eds., Elsevier: Netherlands, 2017; 163–170. 0.1016/j.trpro.2017.05.084.

[35] Jolliffe I.T., Cadima J., Principal component analysis: a review and recent developments Phil. Trans. R. Soc. 2016 A.37420150202; 10.1098/rsta.2015.0202. PMC4792409 26953178

[36] United Nations, Transforming our World: The 2030 Agenda for Sustainable Development, 2016. Available online: https://sustainabledevelopment.un.org/ (accessed on 11.03.2020).

[37] Hagai K.; Keck W.M. Independent factor analysis. *Neural Computation—NECO*, 1999, 11(2), 803–851. 10.1162/089976699300016458 10226184

[38] Everitt B.S.; Landau S.; Leese M.; Stahl D. *Cluster Analysis*, 5^th^ ed; John Wiley & Sons 2011; ISBN. 9780470749913.

[39] Salvati L., Zambon I., Chelli F. M., Serra P. Do spatial patterns of urbanization and land consumption reflect different socioeconomic contexts in Europe?. *Science of The Total Environment*, 2018, 625, 722–730; 10.1016/j.scitotenv.2017.12.341 29306160

[40] Yan Y.; Wang Ch.; Quan Y.; Wu G.; Zhao J Urban sustainable development efficiency towards the balance between natural and human well-being: Connotation, measurement and assessment. *Journal of Cleaner Production*, 2018, 178, 67–75; 10.1016/j.jclepro.2018.01.013

[41] Gonzalez-Garcia S.; Manteiga R.; Moreira M.T.; Feijoo G. Assessing the sustainability of Spanish cities considering environmental and socio-economic indicators. *Journal of Cleaner Production*, 2018, 178, 599–610; 10.1016/j.jclepro.2018.01.056

